# A Spatio-Temporal Entropy-based Framework for the Detection of Trajectories Similarity

**DOI:** 10.3390/e20070490

**Published:** 2018-06-23

**Authors:** Amin Hosseinpoor Milaghardan, Rahim Ali Abbaspour, Christophe Claramunt

**Affiliations:** 1 School of Surveying and Geospatial Engineering, College of Engineering, University of Tehran, 1439957131 Tehran, Iran; 2 Naval Academy Research Institute Lanveoc-Poulmic, BP 600, 29240 Brest Naval, France

**Keywords:** trajectory, Spatio-temporal entropy, similarity

## Abstract

The rapid proliferation of sensors and big data repositories offer many new opportunities for data science. Among many application domains, the analysis of large trajectory datasets generated from people’s movements at the city scale is one of the most promising research avenues still to explore. Extracting trajectory patterns and outliers in urban environments is a direction still requiring exploration for many management and planning tasks. The research developed in this paper introduces a spatio-temporal framework, so-called STE-SD (Spatio-Temporal Entropy for Similarity Detection), based on the initial concept of entropy as introduced by Shannon in his seminal theory of information and as recently extended to the spatial and temporal dimensions. Our approach considers several complementary trajectory descriptors whose distribution in space and time are quantitatively evaluated. The trajectory primitives considered include curvatures, stop-points, self-intersections and velocities. These primitives are identified and then qualified using the notion of entropy as applied to the spatial and temporal dimensions. The whole approach is experimented and applied to urban trajectories derived from the Geolife dataset, a reference data benchmark available in the city of Beijing.

## 1. Introduction

Trajectory data often available in modern cities offers many opportunities for the analysis of human movement required by urban management and planning tasks. Cities are complex and dynamic environments whose underlying human behaviors and displacements are far from being straightforward processes to model and understand. At the primitive level, a trajectory can be considered to be the basic displacement in space and time of one or many human beings. A trajectory can be modeled as a series of line segments from a starting point to an ending point. When aggregated, trajectories exhibit patterns whose distribution in space and time can be analyzed at different levels of scale and temporal granularity, from the understanding of traffic patterns to the analysis of human behaviors in the city [1]. Indeed, behavioral studies require the combination of not only trajectories considered to be geometrical data but also additional semantics that will provide additional insights on the reasons and motivations behind these displacements [2]. However, and in the context of this paper, we postulate that, while limited to the spatial and temporal patterns that emerge from large trajectory datasets, large trajectory datasets generated in urban environments are likely to provide useful insights towards a better comprehension of human displacement patterns in the city.

A specific objective of our study is to identify a series of trajectory parameters at the primitive level that will support the identification of similarities in the temporal and spatial dimensions as suggested in [3]. A key question to start with is the prior identification of what kind of primitives one should evaluate when searching for trajectory similarities and differences. Our focus is on the underlying and intrinsic trajectory characteristics in space and time. In other words, two given trajectories will be considered to be relatively close in space, in time and in space and time when the distribution of their underlying properties match. As we would like to evaluate such properties in the spatial and temporal dimensions, we consider a trajectory as a primitive abstraction whose internal properties will be cross-compared to potentially similar ones. This leads us to consider not starting and ending points as well as trajectory direction as explored in related work (e.g., [4]), but rather a series of qualitative parameters such as curvatures, stop points, turning points and self-intersections. Typical time parameters such as velocity and acceleration as studied in some related work (e.g., [5]), and metrics in the spatial dimension will be considered to be embedded in the respective temporal and spatial dimensions and therefore implicitly considered by our approach.

So far, current methods oriented to the study of trajectory similarities can be categorized into two groups. A first category applies geometrical distances between some given trajectories, that is, cross-calculating and aggregating distances between intermediate points [4]. This can be also evaluated at the segment level according to some given thresholds [6], or in relation to a median trajectory when analyzing a set of trajectories. Despite the interest of these methods, they suffer from several drawbacks: first there is no semantic classification between the intermediate trajectory points considered, next the data volumes generated often lead to heavy computational times. Finally, most of the methods and algorithms developed so far do not consider the temporal dimension closely associated to the intermediate points of the considered trajectories.

The research developed in this paper introduces a novel approach, so-called STE-SD (Spatio-Temporal Entropy for Similarity Detection), that integrates the notion of trajectory critical points and semantic descriptors as well as the spatial and temporal dimensions. The main principle is as follows. First, critical points are detected and identified according to some geometric and semantic parameters. Next, we apply a series of entropies concept derived from the theory of information as introduced by Shannon [7]. The motivation behind the application of several entropy measures is as follows. The main idea is to evaluate the internal diversity of the geometrical and semantic parameters of a given trajectory, and thus according to some spatial, temporal, and spatio-temporal parameters. Such diversity should reflect the relative importance and distribution of the different geometrical parameters identified for each trajectory, and consider the respective spatial and temporal distribution of the different parameters identified. Another potential advantage of the proposed method is the possibility of comparing different parts of two trajectories based on different spatial and temporal entropy parameters. The rest of the paper is organized as follows. Section 2 briefly surveys related work while the main principles of our approach are introduced in Section 3. Section 4 develops an experimental evaluation. Finally, Section 5 concludes the paper and outline further works.

## 2. Related Work

Current approaches can be categorized according to the following principles and categories (Table 1):

- semantic-based algorithms oriented to the detection of points of interest, starting and ending trajectory points. The main objective of these methods is to identify the most relevant semantic characteristics of the trajectories studied (e.g., frequent stops) [4,8,9];
- spatio-temporal data mining and analysis approaches based on spatial, temporal and spatio-temporal functions and clustering analysis. Their objectives are oriented to the detection of movement patterns, trajectory similarities and outliers [10,11,12]. Such approaches have been particularly applied to derive behavioral patterns. Different criteria can be applied from distance-based measures [13,14] to the application of clustering algorithms [15,16,17];
- graph-based measures and algorithms where node-edge connectivity are analyzed at large according to some spatial and temporal criteria such as displacement times, regularities and irregularities [18,19].

The above classification is refined by a second one introduced in Table 2, where most of categories are specified according to a series of spatial, temporal and semantic parameters. Some of the approaches privilege geometrical properties such as series of trajectory intermediate points, different measures of distance [2,52] and/or additional measurements such as curvature, direction and sinuosity. Others also integrate space and time when considering velocity and acceleration. Last external factors such as environmental conditions have been also considered [21,36].

Most of the methods mentioned above share the fact that they are applied to very large sets of trajectories and to incoming trajectories in their most extended form, that is, all intermediate trajectory points are considered in the process. While this has the advantage of keeping all the incoming trajectory properties, this has the disadvantage of increasing the complexity of the methods applied and computational execution times. This leads us to suggest and develop an alternative approach whose objective will be to identify the trajectory critical points, that is, the ones that embed some relevant spatial, temporal and semantic properties that should be kept to reflect the specific peculiarities of the trajectory considered. In fact, the detection of trajectory critical points has been already mentioned as a key issue [31,42] in many related graph-based and Origin-Destination methods, while in these later methods intermediate points of interest are not considered. Our approach will consider simultaneously all the spatial, temporal and semantic dimensions within an integrated framework. Next, we will introduce several measures of entropy that will provide a quantitative evaluation of the qualitative properties that emerge from the spatial, temporal and semantic dimensions considered within an integrated framework. The whole approach is based on a series of spatial and temporal entropy measures introduced in previous works [53,54,55,56,57].

## 3. Methodology

**Figure 1:**
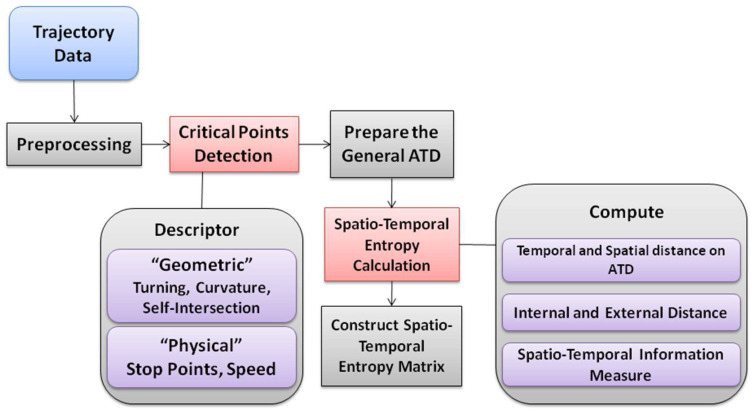
Method flowchart.

The general implementation steps of the proposed method are shown in Figure 1 as a flowchart. The framework includes two main steps.

1. Detection of the critical points according to some predefined parameters.
2. Spatial and temporal entropy calculation.

To keep track of the most representative trajectory points, a pre-processing step is applied. The objective behind is to eliminate noisy data due to some positioning mismatch or errors. A Kalman Filter is applied in a sort of recursive algorithm by considering a covariance matrix of a series of points. A simple linear model is applied to determine the first line of each trajectory. Therefore, the variance (σ) of the distance between two consecutive points in a given trajectory is calculated, and the points with greater distance than (2σ) from the fitting line are detected as noisy data and eliminated.

### 3.1. Critical Points Detection

The first methodological objective is the derivation of a minimum number of critical points according to the geometric and semantic characteristics of some trajectory data. Extra trajectory data not specifically relevant and noisy will be eliminated to facilitate further processing times. The geometric parameters considered include curvature, turning and self-intersection points. They are detected by the application of a convex hull structure as introduced in our previous work [58]. More precisely, these three geometric parameters can be defined as follows:

- **Curvature:** a curvature denotes a curve in a trajectory and a curvature point is defined as the central point of the curve.
- **Turning point**: a turning point is considered to be a changing direction point of a trajectory. For instance, a straight trajectory with no curve does not have any turning points while two turning points arise for each trajectory curvature.
- **Self-intersection point**: a self-intersecting point denotes a point where the trajectory passes through at least twice. A self-interacting point is considered to be a critical point as it encompasses some specific important properties: a self-intersecting point in a trajectory represents a node surely denoting the relative importance of that self-intersecting point. Among different parameters that can be considered for qualifying a self-intersecting point, the time and distance covered by the self-intersecting part of the trajectory between passing through twice can be mentioned.

Trajectory Critical Points are detected by the application of a Convex-Hull algorithm (TCP-CH) that has been applied to detect the main geometric characteristics, that is, curvature, turning and intersection points as introduced in our previous work [58]. Prior to the application of the TCP-CH algorithm, noisy points are removed by the application of a Kalman filter whose objective is to smooth the successive positions by recursively correcting error values often generated by GPS positioning errors. Curvature and turning points are identified by the TCP-CH algorithm in such a way that starting and ending points of the convex hulls are selected as turning points, while for each convex hull the curvature point is the one located at the maximum distance from the connecting line of two turning points [58].

Additional spatio-temporal properties are derived such as velocity changes and acceleration peculiar behaviors. Additional semantic descriptors are stop points as these are often connected to some worthwhile activities or bottlenecks. Stop-points are identified by the application of Dempster-Shafer theory and application of belief and non-belief functions [59]. Besides the detection of these stop points, the uncertainty of the detection process is also considered. Overall, the velocity is derived for all relevant trajectory points. Therefore, without loss of generality, we introduce the following rule to identify a velocity change at a given trajectory point:

**Rule** **1**. *There is a velocity change at the trajectory point t_i_ of a trajectory T if and only if:*

(1) $$V\left(t_{i}\right)>\beta \mu_{i}\;where\;\mu_{i}=\frac{1}{n}\sum_{j=i-10}^{i}V\left(t_{j}\right)$$

*where T(t_i_) and V(t_i_) respectively represent the position and velocity of a trajectory point t_i_, µ_i_ the velocity average for the trajectory points t_i,_ t_i−1, …,_ t_i−10_, β a parameter that denotes the expected magnitude of the velocity change (e.g., valued as 4/3 in the experiments developed).*

**Figure 2:**
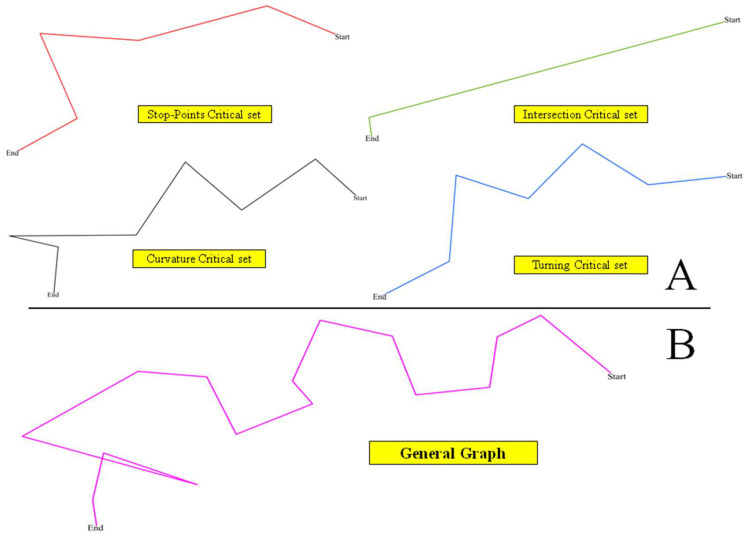
Critical points and ATD representation of a given trajectory example. (**A**) ATD of physical and geometric descriptors; (**B**) General trajectory.

All the parameters listed above as well as the given times of the trajectory points denoted as Time *(t_i_)* are detected and stored in a series of Abstract Trajectory Descriptor (ATD) representations that also consider the starting and ending points, as well as the critical points identified by the geometrical and semantic-based parameters. Figure 2A illustrates the principles behind the different trajectory critical points (i.e., self-intersections, direction changes, curvatures, stop points) and how such ATDs should be derived. Figure 2B gives a schematic representation of the trajectory according to these critical points. The main principle behind this approach is to favor a comparison of several trajectories according to the parameters identified, and considering the underlying spatial and temporal properties embedded in these critical points.

### 3.2. Spatio-temporal Entropy Approach Principles

The STE-SD (Spatio-Temporal Entropy for Similarity Detection) approach developed is based on an application of the entropy theory which has been initially proposed for by Shannon as a probability distribution function to measure the data distribution in a given set of categorical data [7]. The reasons behind this choice and application of the concept of entropy can be given according to the following motivations:

- Not only the usual spatial distribution of the intermediate trajectory points should be considered, but also their temporal distribution. Next, additional parameters such as velocity should be considered in order to reflect some intrinsic changes in the trajectory.
- Trajectories encompass some specific behavior reflected by critical points as identified by our approach (stop points, direction changes, curvature, self-intersections). Such critical points embed some spatial, temporal and semantic properties that represent some very specific behaviors that are crucial when cross-comparing several trajectories.
- The reasons mentioned above leads us to search for and apply a measure of entropy that will reflect the spatial, temporal and semantic distribution of all these parameters within the intimate representation of some trajectories. The objective is to provide a sort of quantitative evaluation of such qualitative properties that will help to evaluate trajectory similarities and differences, and thus according to different configurationally parameters (as another objective is to provide a flexible evaluation approach).

While the measure of entropy as introduced by Shannon has been largely applied to the distribution of categorical data, it must be extended to the spatial and temporal dimensions to take into account the very peculiarities of space and time, and how the different parameters identified are related in space and time [60]. In fact, we look forward a measure of entropy in space and time that can characterize the distribution and diversity of critical points embedded in a given trajectory. In a previous work, a related idea has been applied in Regional Science where a density-based analysis of the distribution of some entities has been developed [61]. Nevertheless, what was required for this study is a comprehensive method that can overall consider the spatial and temporal distances among such critical points. To do so we applied a concept of spatio-temporal entropy introduced in a related work [62]. The main idea of this notion of spatio-temporal entropy is derived from the first law of geography that states that “*Everything is related to everything else but near things are more related than distant things*” [63]. The different measures of spatio-temporal entropies suggested thus consider that the diversity of a given distribution in space and time will increase when different things are closed, whole the diversity will also increase when similar things are distant. The next section will develop the way the measures of spatio-temporal entropies can be applied to our critical-point-based representation of a trajectory.

### 3.3. Spatio-temporal Entropies

This goal of this section is to present how the concepts of spatial and temporal entropies can be applied to trajectory data according to the different measures of distances and critical points considered. As mentioned in the previous section, and as introduced in [62] for a given configuration, the entropy value will increase when the distance between similar entities increases, or when the distance between different entities decreases. Accordingly, two concepts of internal and external distances have been suggested to derive such measure of spatial entropy [62]. The *inner distance* of a class *j* denoted by *d_j_* gives the average of the distances between the entities of this class *j*. Similarly, the *external distance* denoted by *d_j_* represents the average of the distances between the entities of this class *j* and the other entities that do not belong to this class *j*. These respective inner distance (2) and external distances (3) are given as follows:

(2) $$d_{j}^{int}=\frac{1}{N_{j}\times \left(N_{j}-1\right)}\sum_{\begin{array}{c} i=1\\ i\in C_{j} \end{array}}^{N_{j}}\sum_{\begin{array}{c} k=1\\ k\neq i\\ k\in C_{j} \end{array}}^{N_{j}}d_{i,k}\;if\;N_{j}>1$$

(3) $$d_{j}^{ext}=\frac{1}{N_{j}\times \left(N-N_{j}\right)}\sum_{\begin{array}{c} i=1\\ i\in C_{j} \end{array}}^{N_{j}}\sum_{\begin{array}{c} k=1\\ k\notin C_{j} \end{array}}^{N-N_{j}}d_{i,k}\;if\;N_{j}\neq N$$

where *c_j_* represents the set of entities of class *j*, while *n_j_* represents the cardinality of *c_j_*. *N* is the total number of entities, *d_i,j_* represents the distance between entities *i* and *j*.

To apply the principles of the *inner distance* and *outer distance*, let us consider the spatial distance $d_{i,k}^{T}$ between two critical points *t_i_* and *t_j_* of a given trajectory *T*. Hereafter, geometric and semantic descriptors introduced in the previous section are considered to be separate classes. Similarly, critical points are considered to be specific classes. This measure of spatial distance, as applied to the critical points of trajectory data, supports derivation of internal and external distances, and this for all classes of semantic and geometric descriptors. Let us therefore introduce the measure of spatial entropy as introduced in [62], and as based on the measures of internal and external distances:

(4) $$H_{s}=-\sum_{i=1}^{n}\frac{d_{i}^{int}}{d_{i}^{ext}}p_{i}log_{2}\left(p_{i}\right)$$

where *H_s_* is the measure of entropy, *p_i_* is the percentage of entities in the class *i*.

To apply this measure of spatial entropy we first evaluate the internal distance distribution of the critical points of a given class, and secondly the external distribution of the critical points of a given class with respect to the other critical points quantified by the measure of external distance. This will further provide a valuable direction to explore and quantify the possible similarities of different trajectories, and this according to some predefined critical points that encompass some specific semantic. For instance, curvature points reflect some specific properties of a given trajectory that can be cross-compared across several trajectories. Similarly, direction changes or self-intersections represent some specific behaviors when a human being is for example acting and moving in a given urban environment (or an animal behaving in a natural environment). Distances between such critical points often represent specific patterns. The frequency of the appearance of the different classes of critical points is another noteworthy trend to consider. These few examples show the variety of potential approaches for comparing the different semantic and spatio-temporal properties closely associated to some given trajectories. This is the reason for suggesting a flexible spatio-temporal measure of entropy that can outline such properties in different ways. The other option retained is to evaluate the ratio between the internal and external distances as given in Equation (4).

Similar to the way the measures of inner distance and outer distance have been applied to the spatial dimension, the measures of *internal temporal distance* (5) and *outer temporal distance* (6) have been also introduced to support the definition of a notion of temporal entropy [62]:

(5) $$td_{j}^{int}=\frac{1}{N_{j}\times \left(N_{j}-1\right)}\sum_{\begin{array}{c} i=1\\ i\in C_{j} \end{array}}^{N_{j}}\sum_{\begin{array}{c} k=1\\ k\neq i\\ k\in C_{j} \end{array}}^{N_{j}}td_{i,k}$$

(6) $$td_{j}^{ext}=\frac{1}{N_{j}\times \left(N-N_{j}\right)}\sum_{\begin{array}{c} i=1\\ i\in C_{j} \end{array}}^{N_{j}}\sum_{\begin{array}{c} k=1\\ k\notin C_{j} \end{array}}^{N-N_{j}}td_{i,k}$$

where $td_{j}^{int}$ and $td_{j}^{ext}$ respectively represent the internal and external temporal distances of the class *j*, respectively, and where $td_{i,k}$. represents the temporal distance between two entities *i* and *j* as follows: $td_{i,j}^{T}=t_{j}^{T}-t_{i}^{T}$.

Next, the temporal entropy $H_{T}$ for each trajectory *T* is computed as follows [62]:

(7) $$H_{T}=-\sum_{i=1}^{n}\frac{td_{i}^{int}}{td_{i}^{ext}}p_{i}log_{2}\left(p_{i}\right)$$

where *p_i_* denotes the ratio of the critical points of the class *i* over all the classes.

The next goal is to introduce a spatial-temporal entropy matrix for all relevant trajectory data. The interest of this matrix is to give an overview of the different measures applied for a given set of trajectories. The dimensions of this matrix are ((2*m +* 2) × *n*) where *n* denotes the number of trajectories considered, and *m* denoting the total number of semantic and geometrical parameters, and *T*_1_, *T*_2_,…, *T_n_* represent the id of trajectory 1, trajectory 2 and trajectory 2, respectively. Table 3 presents the general structure of this matrix and the different critical points considered for each trajectory.

Finally, the measure of Spatio-Temporal entropy is computed as follows [62].

(8) $$H_{ST}=-\sum_{i=1}^{n}\frac{std_{i}^{int}}{std_{i}^{ext}}p_{i}log_{2}\left(p_{i}\right)$$

with

(9) $$std_{i}^{int}=d_{j}^{int}\times td_{j}^{int}$$

(10) $$std_{i}^{ext}=d_{j}^{ext}\times td_{j}^{ext}$$

**Figure 3:**
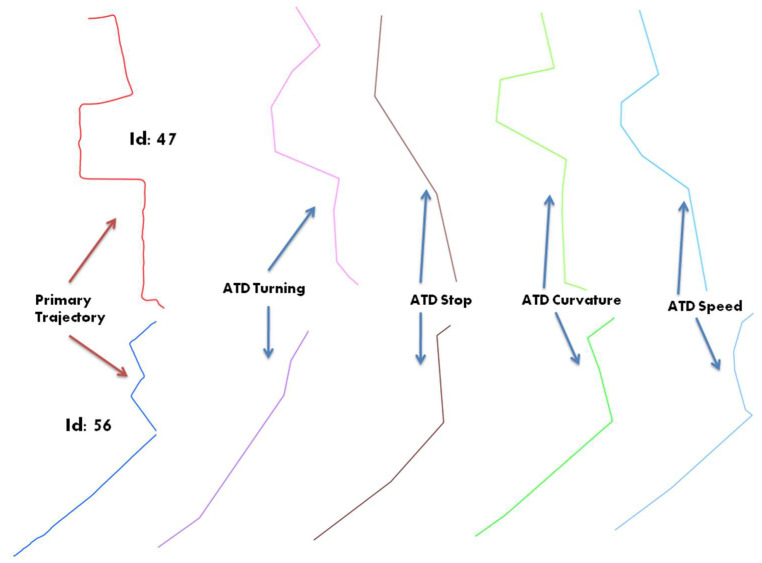
Two trajectories 47 and 56 with their derived ATD representations.

And where $H_{ST}$, $std_{i}^{int}$. and $std_{i}^{ext}$ denote the spatio-temporal entropy, spatio-temporal internal and external distances, respectively. Based on the entropy derivations introduced in Table 3, each trajectory can be qualified using different spatial and temporal descriptors. Indeed, and depending on the application context, one might either consider some geometrical primitives such as curvature, turning or intersection points, or rather some spatio-temporal behavior such as trajectory speed changes. Let us for instance consider two trajectories extracted from the sample data and shown in Figure 3. This schematic representation illustrates how these trajectories can be abstracted by considering different primitives, and then generating a sort of derived ADT trajectory representation.

As derived towards an ADT representation, each trajectory can be qualified by applying the measures of spatial and temporal entropies as presented in Table 4.

The results presented in Table 4 can be used to describe the semantics embedded in the trajectories 47 and 56, and for comparing them according to some preselected descriptors. For example, the results above outline a close similarity when considering the temporal dimension rather than when considering the spatial dimension.

## 4. Experimental validation

This section reports on the experiments developed so far. We first briefly introduce the reference datasets used, and then discuss the different applications of the spatial-entropy measures. The implications of the whole findings are then discussed.

### 4.1. Reference Data

**Figure 4:**
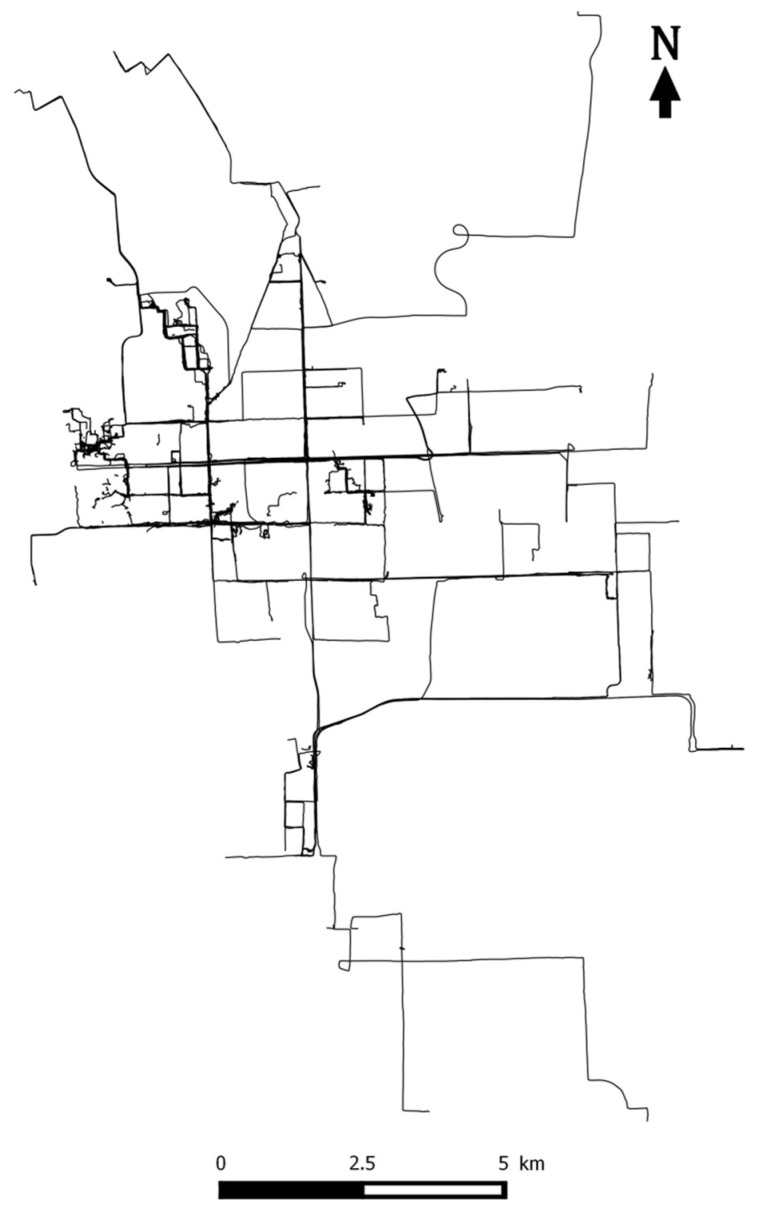
Selected 326 trajectories for implementation.

The principles of our approach are applied to a large urban trajectory dataset available in the city of Beijing. The Geolife project collected a large repository of urban trajectories recorded by taxis, buses or even human beings equipped with GPS receivers from 2007 to 2012 [21]. The main advantage of this reference dataset is that it is fully available and has been largely used since as a benchmark database for further research and studies. From the initial 326 trajectories we selected a sample that reflects a relatively large variety of human displacements performed either by taxis, buses or even walking as presented at Figure 4. There is also no restriction for a user to perform several trajectories. These trajectories overall represent 83,412 trajectory points and a total distance of 672,195 m. The shortest trajectory covers 8.54 m while the longest one covers 14,408.2 m; the mean length of these trajectories is 2417.97 m. Likewise, the mean sampling distance covered between two trajectory points is 10.21 m and the mean sampling time is 5.11 s.

### 4.2. Implementation

According to the principles of the approach introduced in the previous sections, the STE-SD method involves three general steps. At first, Convex Hulls structures implemented as a computational method, a preprocessing phase is applied to get rid of unnecessary points. Next, critical points are identified for each semantic and geometric descriptor. Finally, the different measures of spatio-temporal entropies are derived and summarized according to the principle of the matrix introduced in the previous section.

#### 4.2.1. Critical Points Detection

##### Convex Hulls

**Figure 5:**
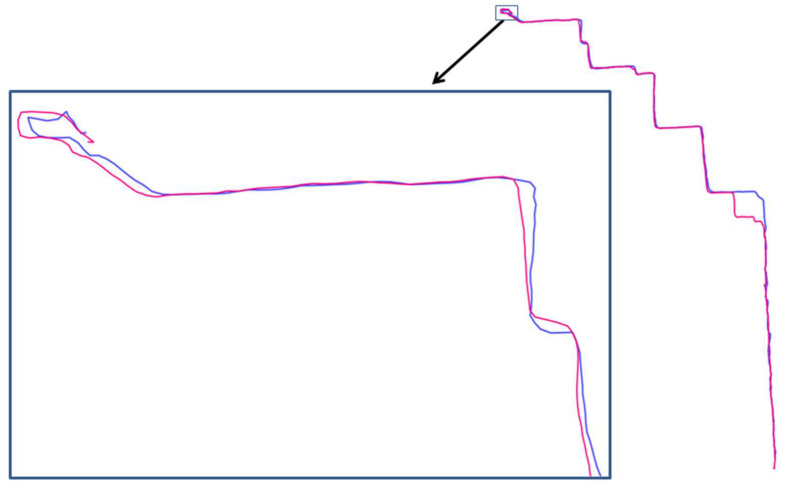
Two similar trajectories but with different CHs. Two different trajectories presented by Blue and purple colors.

Convex hulls have been derived for the 326 trajectories and by application of an algorithm introduced in our previous work [58]; a total number of 7498 convex hulls were obtained. Convex hulls are particularly useful when detecting light differences between similar trajectories according for instance to their origin and destination. This is the case for the example presented in Figure 5 as trajectory id 72 encompasses 118 convex hulls while trajectory id 75 with 125. While the two trajectories presented in Figure 5 have relatively similar paths, and at the local scale, they exhibit some large differences regarding the number of convex hulls. Different sampling times and an insufficient prior cleaning process are likely to explain these differences. In particular, the critical points identified for these convex hulls give additional insights when analyzing trajectory differences and similarities.

**Figure 6:**
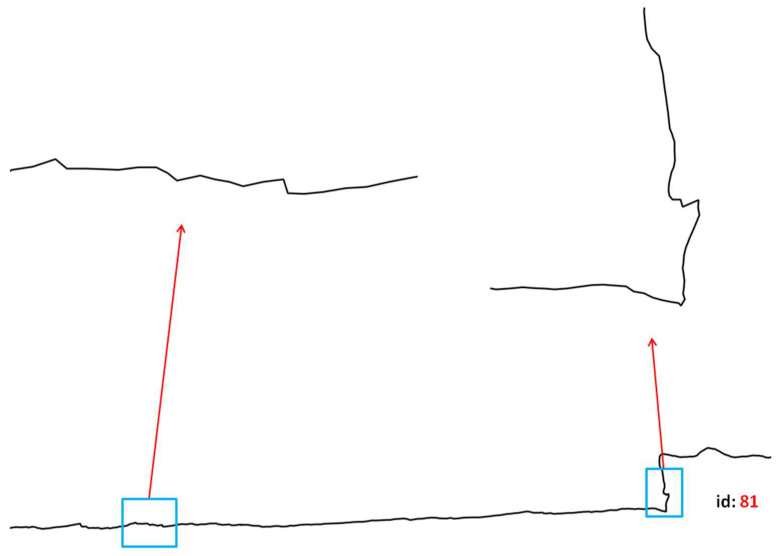
Impact of the Convex Hull threshold on a trajectory.

Next, by detecting curvature point, small and therefore noisy convexes are removed by defining a threshold of 0.02D (where D denotes the length of the trajectory), that is, curvatures with lengths lower that this threshold value are removed. To find the best balance between the need to keep the main semantics of a given trajectory while reducing its complexity, the most appropriate threshold value should be identified. In the context of the dataset used by the experimental validation, it appears after several iterations that a threshold value of 0.02 is appropriate to delete noisy convex. This threshold value provides a good compromise between the need to delete small convex and the necessity of preserving large ones. Let us consider two convex parts of the trajectory id 81 shown in Figure 6. Application of the considered threshold removes the small convex to the left, while conserving the more significant one to the right.

Accordingly, 2317 Convex Hull structures were removed from the initial set of convex hulls. Table 5 summarizes the number of deleted convex hulls and critical points obtained in our trajectories sample. Figures are classified according to the trajectories length. 

The results presented in Table 5 show the main trends of our data sample regarding the main trajectory characteristics identified so far. In particular, it clearly appears that the most curved trajectories are the ones of the 3rd category as well as the ones that have the higher number of removed small curves.

##### Speed Change Points

**Figure 7:**
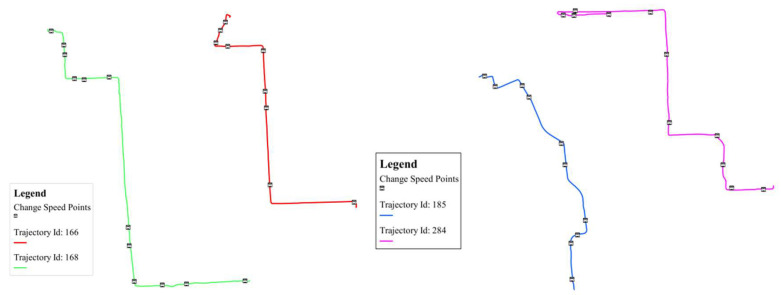
**Left**: similar geometries with different speed behaviors; **Right**: different geometries with similar speed behaviors.

By the applications of Equation (1) introduced in Section 3.1 critical points that denote speed changes were identified. Indeed, and as illustrated in Figure 7, these critical points do not have a direct relationship with the intrinsic trajectory geometry. For example, Figure 7 left shows a trajectory with different geometrical and speed change patterns, while on the contrary Figure 7 right shows two different trajectories with relatively similar speed change patterns. This might support the cross-comparison of behavioral patterns across different trajectories, as well as comparing the geometrical and spatio-temporal patterns that emerge for a given trajectory.

**Figure 8:**
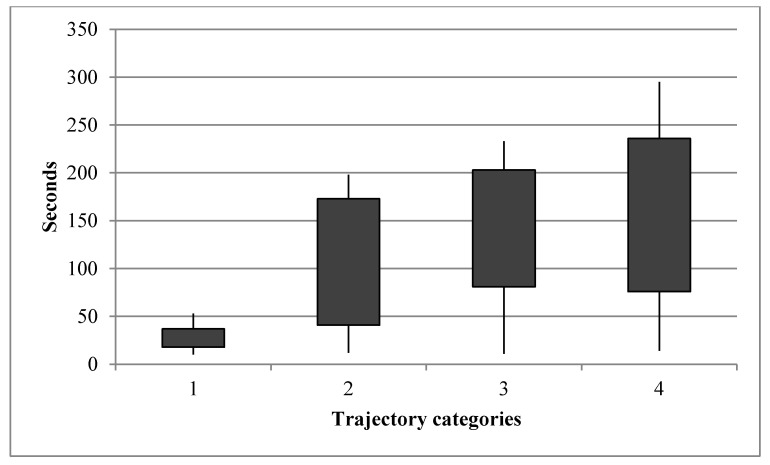
Box Plot graph of the distribution of temporal distances between speed-change points.

Overall, the number of detected speed changes point in the trajectories sample is 5720. An interesting property and pattern to highlight is how often there is a speed change in a given trajectory. We denote such time intervals and summarize them in Figure 8.

Figure 7 clearly reveals that there is a direct relationship between the time distances between speed-change points and the respective lengths of the trajectories.

##### Stop Points

The detection of stop points is far from being a straightforward task when especially considering the notion of uncertainty. In a related work we introduced an approach based on the Dempster-Shafer theory of evidence, and whose objective is to detect trajectory stop points and associated degrees of uncertainty [59]. The principles behind this approach is that for each candidate stop point, the Belief, Disbelief and Uncertainty values are derived, and the movement status of the points (i.e., stop or moving) is determined. The results show high precision when detecting stop points as compared to previous approaches. In particular, the experimental evaluations indicate that the mean uncertainty overall associated with the detected stop points, in comparison with similar extracted points by the other methods, is much lower and meanwhile possesses a higher Belief value. This outlines the important role of uncertainty in the results and the efficiency of the Dempster-Shafer theory of evidence when applied to this case. The minimum, maximum and average values of the Belief, Non-belief and uncertainty of all trajectory points including the identified stop points are given in Table 6.

#### 4.2.2. Spatial and Temporal Entropy

To derive spatial and temporal entropy values, the respective internal and external temporal and spatial distances between the identified critical points of each trajectory are derived for the selected 326 trajectories, as well as a few additional parameters (Table 7 and Table 8).

This table shows that overall the average of spatial distances between consecutive critical points when considering semantic parameters is higher than for geometrical parameters. This is a worthwhile pattern to highlight: critical points identified in the semantic dimension are less likely to be closer than the ones identified in the spatial dimension. Figures that characterize temporal distances between successive critical points for the aforementioned parameters are presented in Table 8.

Next, both internal and external spatial and temporal distances were derived (Table 9). A series of valuable trends appear. First internal distances are generally lower than external distances, this denoting the fact that for a given category of critical points, other critical points from the same category are generally close that the ones of different category. It also appears that this trend is much more significant in time than in space.

**Figure 9:**
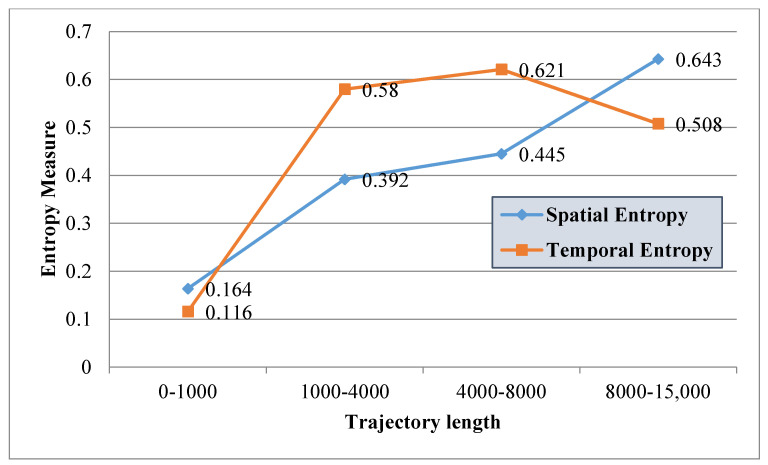
Spatial and temporal entropies derived for the sample trajectories.

Next, spatial and temporal entropies are derived for each trajectory using Equations (10) and (11). The mean values of the spatial and temporal entropies for all 326 trajectories categorized according to length criteria are presented in Figure 9.

**Figure 10:**
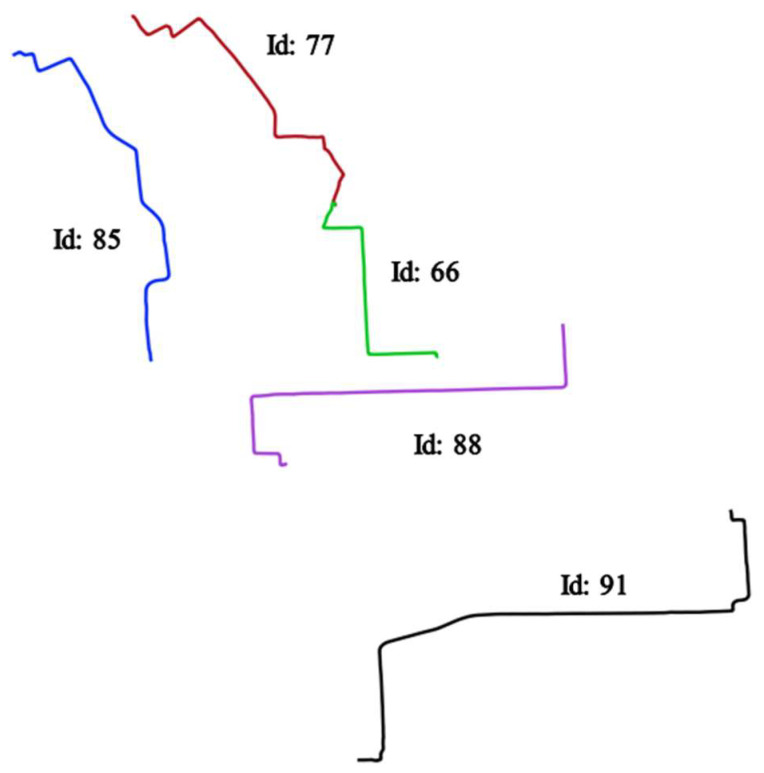
Selected trajectories for the entropy evaluation.

Overall it appears that the longer a trajectory, the higher the spatial entropy will be. Regarding the temporal entropies, it also appears that longer trajectories have relatively higher entropy values but not with a linear increase as for the spatial entropy. This outlines the fact that these two measures are not completely correlated and therefore surely complementary indices when the objective is to analyze the distribution of the structural properties of a trajectory. An interesting trend that appears is that there is a decrease in temporal entropies for the longest trajectories. In fact, the longest trajectories often have a lower shape complexity thus also generating a significant increase of external distances and to a lower degree a decrease of internal distances. Figure 10 illustrates the outputs for 5 different trajectories. Trajectories 66, 88, and 91 have a similar geometry but different directions, while trajectories 77 and 85 have different geometries and directions.

Spatial and temporal entropies for these trajectories are presented in Table 10 and Table 11 (spatial entropies are derived for each category and then aggregated).

**Figure 11:**
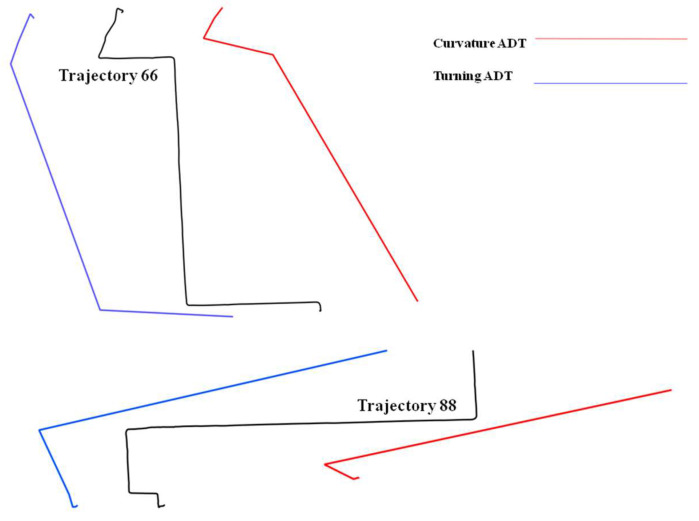
Trajectories 88 and 66 with related curvature and turning ADTs.

The results presented in Table 10 and Table 11 outline different patterns. First it appears that trajectories 66, 88 and 91 have relatively close spatial entropies (respectively 0.86, 0.81 and 0.78) while trajectories 77 and 85 form another similar group with similar entropies (respectively 0.53 and 0.44). Figure 11 illustrates the reason behind the similarity that appears between trajectories 66 and 88. While directions are different, an ADT representation applied to curvature and turning critical points show that the patterns exhibited by these to trajectories are relatively similar, thus explaining the relative close spatial entropies for these two trajectories.

A close examination of the entropy valued exhibited in Table 11 shows a weak relationship with the patterns that appear with the spatial entropies. While trajectories 77 and 85 are relatively similar according to their temporal entropies this is not the case for trajectories 66, 88 and 91. This emphasizes the role of the temporal dimension when analyzing trajectory patterns: similarities in space do not always mean that such similarities will still apply when integrating the temporal dimension.

Next, and to provide another comparison with some additional common geometrical parameters, Table 12 summarizes two geometrical properties derived for these trajectories, that is, shape and complexity. The upper and lower triangle of presented matrix in Table 12 present a one-to-one geometric comparison of these trajectories according to their shape and complexity similarities as introduced in [64]. Shape evaluates the length and angle of turning at each node using specified function as signature function while complexity is derived using the average distance of each node from the connecting line between start and end nodes.

A final comparison of the figures highlighted in Table 12 with the spatial and temporal entropies presented in Table 10 and Table 11 give the following trends and differences:

- According to the spatial and temporal entropies, as well as for the intrinsic geometrical parameters derived from shape and complexity, trajectories 66, 88 and 91 are relatively similar.
- A similar pattern appears for trajectories 77 and 85.
- Conversely, there is no evidence of similarity in the temporal dimension according to the values exhibited by the temporal entropies. This in fact denotes a valuable trend: exhibiting some spatial and geometrical similarities does not imply a similar trend when considering the temporal dimension.

**Figure 12:**
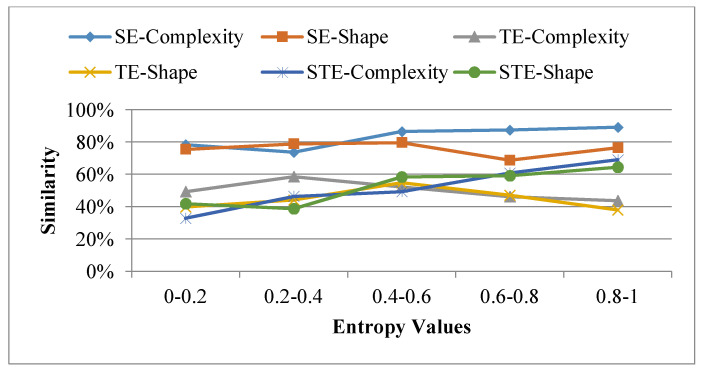
Similarities between geometrical properties and entropies.

To provide a generalization of the specific trends and patterns revealed when considering an arbitrary subset of trajectories, a generalization of the parameters considered has been performed for the whole set of 326 trajectories, and thus for the five different measures considered (shape, complexity, temporal entropy, spatial entropy). Figure 12 shows some evidence of similarities in the spatial dimension when considering purely geometrical of spatial measures of entropy while indeed there is much less similarities between geometrical properties and critical points when considering time and temporal entropies.

Figure 12 summarizes the main similarity patterns that emerge when comparing geometrical and entropy similarities (where SE, TE and STE respectively denote spatial, temporal and spatio-temporal entropies). The main point is that there is an evidence of lack of correlation between geometrical and entropy similarities, the trend being even stronger when considering temporal entropies. Overall this shows the interest of combining geometrical and entropy evaluations when studying some possible similarities and differences when analyzing the possible patterns that emerge from large trajectory datasets.

**Figure 13:**
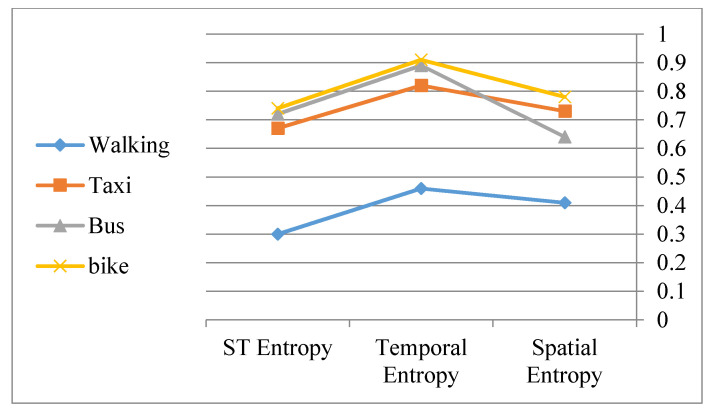
Entropy patterns when considering moving modes.

Lastly, when considering the transportation modes used by the 326 sample trajectories several worthwhile patterns emerge. While taxi, bus and bike displacements show closely related patterns, walking displacements clearly exhibit different patterns and a lower diversity of patterns. (Figure 13).

## 5. Conclusions

The availability of large trajectory datasets in urban environments offers many new opportunities for analyzing human displacements in space and time. Although relatively basic geometrical primitives, trajectories can exhibit some more complex properties when considering their internal structure when considered specifically or as whole semantic, spatial and temporal properties. Indeed, the application domain clearly should play a major role on the way the different representations of a given trajectory, the selection or not of the different spatial and temporal primitives, and finally the way the different diversity and entropy measures should be applied. The main idea behind this approach is to evaluate the internal diversity of the geometrical and semantic parameters of a given trajectory, and thus according to some spatial, and temporal parameters. The diversity measures that appear reflect the relative importance and distribution of the different geometrical parameters identified for a given trajectory. Another potential advantage of the proposed method is the possibility of comparing different parts of two trajectories, based on different spatial and temporal entropy parameters, or by comparing different trajectories. Another option offered by this approach is to apply it for clustering and pattern detection. The research presented in this paper introduced a Spatio-Temporal Entropy computational method (STE-SD) that considers trajectories as structural primitives according to the semantic, spatial and temporal dimensions. The main advantages of the proposed method are summarized below:

- Similarities and differences are analyzed according to the intrinsic spatial and temporal properties of some selected trajectories, and according to a series of geometrical (direction changes, curvatures) and semantic parameters (stop points, speed changes). A peculiarity of the approach is that trajectory similarities are evaluated regardless of the trajectory beginning and end point both in space and time.
- A notion of ATD (Abstract Trajectory Descriptor) is introduced and can be considered to be a sort of geometrical and semantic signature for each considered trajectory. Not only does this provide a structural representation of a trajectory to derive different measures of entropy, but it also gives a simplified representation that is flexible as potentially extendable to take into account additional or different parameters.
- To facilitate the implementation and computation of approach, a prior filtering of the trajectory samples is processed and where critical points only are conserved according to the geometrical and semantic parameters identified and selected.
- The approach is flexible as not only the measures of entropy are derived in the spatial and temporal dimensions, but also as a series of intermediate measures of entropy are derived independently for each identified semantic and geometrical variable. This allows studying at the macro or micro level the diversity of these parameters at different levels.

So far, the whole approach has been applied to a benchmark trajectory database. We plan to apply the proposed method to other urban contexts as well as to different application domains. For example, the analysis of maritime trajectories, or trajectories derived from animal behaviors are among the application directions we plan to consider in our further work. Our research will also be oriented toward the exploration of additional semantic and geometrical parameters as well as computational developments that will improve computational times.

## Figures and Tables

**Table 1:** Classification of current approaches.

Current Approaches	Data Sources			
	GPS Data	Smart Card Data	Cell Phone Data	Other
Semantic-based	[4,8,9,20,21]	[22,23]	[1,24,25]	[26,27]
Spatio-temporal data mining and analysis	[3,5,12,13,14,15,16,17,21,28,29,30,31,32,33,34,35]	[36,37,38,39]	[1,18,40,41,42]	[11,15,43,44]
Graph-based	[45,46,47]	[39,48]	[49,50]	[51]

**Table 2:** Further classification of current approaches.

Properties	Categories	Properties	Related Work
ST data mining and analysis	Spatial	Distance	[3,5,12,13,14,17,18,46,47,49]
		Direction	[1,4,16,17,18,26,28,32,41]
		Turning Angle	[5,11,12,32]
		Sinuosity	[1,11,12,29]
	Spatio-temporal	Velocity	[4,5,11,18,28,47]
		Acceleration	[5,28,29]
	Temporal	Time of Occurrence	[8,9,15,17,22,36,38,42,44,49,50]
Semantic		Environment data	[1,11,15,20,21,28,36,37,38]
		Stop Point	[14,16,17,18,26,28,37,45]
		POI	[1,3,14,21,22,30,31,32,33,36,41,42,43,44,45,46,47,50,51]
		People attributes	[4,20,21,36,37]

**Table 3:** Spatial-temporal entropy matrix.

			*T*_1_	*T*_2_	*T*_3_	…	*T_n_*
**Spatial**	Spatial Entropy		*V*_11_	*V*_12_	.	…	*V*_1*n*_
	Semantic information measure	speed	.				.
		stop	.				.
	Geometric information measure	Curvature	.				.
		Turning	.				.
		Intersection					
**Temporal**	Temporal Entropy						
	Semantic information measure	speed					
		stop					
	Geometric information measure	Curvature	.				.
		Turning	.				.
		Intersection	*V*_61_	.	.	…	*V*_6*n*_

**Table 4:** Entropy descriptors for sample trajectories 47 and 56.

Entropy Type	Trajectory Id	Stop	Speed	Turning	Curvature	Aggregated Entropy
**Spatial**	**47**	0.11	0.34	0. 38	0.53	0.376
	**56**	0.14	0.51	0.46	0.18	0.287
**Temporal**	**47**	0.36	0.29	0.41	0.32	0.335
	**56**	0.28	0.53	0.49	0.27	0.359

**Table 5:** Critical points detection.

Category	Length of Trajectory (m)	N. of Trajectories	N. of Primary CH	N. Removed CH	Variance of Distance to CH Line	N. of Curvature Points	N. of Turning Points	N. of Intersection Points
**1**	0–1000	56	510	63	3.41	447	449	5
**2**	1000–4000	82	1245	235	7.33	1010	1012	26
**3**	4000–8000	127	3910	1376	14.57	2534	2536	58
**4**	8000–15,000	61	1833	639	17.20	1194	1196	31

**Table 6:** Belief, non-belief and uncertainty values for candidate stop points.

	For All Points			For Stop Points		
	Minimum Value	Maximum Value	Average	Minimum Value	Maximum Value	Average
Belief	0.09	0.945	0.864	0.723	0.945	0.834
Disbelief	0.02	0.894	0.448	0.12	0.27	0.145
Uncertainty	0.04	0.23	0.135	0.06	0.19	0.125

**Table 7:** Spatial distances between successive critical points.

Spatial Distances (m)					
	Semantic Parameters		Geometric Parameters		
	Stop	Speed	Turning	Curvature	Intersection
Minimum	81.3	66.9	138.4	185.2	0
Maximum	6590.1	1631.4	650.9	718.1	1903.8
Mean	1073.6	589.3	315.7	377.6	661.5
Variance	456.27	129.43	96.67	89.16	104.17

**Table 8:** Temporal distances between successive critical points.

Temporal Distances (S)					
	Semantic Parameters		Geometric Parameters		
	Stop	Speed	Turning	Curvature	Intersection
Minimum	9	11	16	25	0
Maximum	592	236	51	78	389
Mean	68	125	34	47	235
Variance	21	38	10	17	107

**Table 9:** Internal and external spatial and temporal distance averages.

		Semantic Parameters		Geometric Parameters		
		Stop	Speed	Turning	Curvature	Intersection
Spatial	Internal Distance	1099.7	1425.2	1669.5	1351.8	744.5
	External Distance	1733.9	1886.5	1905.2	1922.8	2185.3
Temporal	Internal Distance	6720	8447	12971	9832	4063
	External Distance	21,849	45,216	66,213	54,470	83,416

**Table 10:** Spatial entropies of the sample trajectories.

Trajectory Id	Length (m)	Spatial Entropies				
		Stop	Speed	Turning	Curvature	Aggregated Spatial Entropy
**66**	5146	0.58	0.65	0.27	0.24	0.76
**77**	6990	0.75	0.24	0.16	0.11	0.46
**85**	8082	0.80	0.26	0.19	0.14	0.52
**88**	9079	0.52	0.69	0.30	0.27	0.81
**91**	11,806	0.44	0.61	0.26	0.23	0.78

**Table 11:** Temporal entropies of the sample trajectories.

Trajectory Id	Length (m)	Temporal Entropies				
		Stop	Speed	Turning	Curvature	Aggregated Temporal Entropy
**66**	5146	0.24	0.47	4.55	4.28	0.74
**77**	6990	0.11	0.63	3.12	2.77	0.77
**85**	8082	0.08	0.69	2.91	2.65	0.69
**88**	9079	0.45	0.63	5.76	3.91	0.91
**91**	11,806	0.32	0.48	7.24	5.39	0.82

**Table 12:** Comparison matrix of sample trajectories using shape (upper triangle) and complexity (lower triangle).

Trajectory No.	66	77	85	88	91
**66**		32.86%	27.11%	79.2%	78.49%
**77**	38.29%		83.26%	42.18%	39.95%
**85**	35.08%	86.4%		37.1%	33.72%
**88**	79.51%	38.48%	41.23%		80.68%
**91**	74.93%	45.76%	38.02%	83.42%	
